# Expand Your Horizon: A Qualitative Analysis of How Adolescent Girls With an Eating Disorder Describe Their Body Functionality

**DOI:** 10.1111/eat.70006

**Published:** 2025-11-18

**Authors:** Stella Weiland, Jessica M. Alleva, Klaske A. Glashouwer

**Affiliations:** ^1^ Department of Clinical Psychology and Experimental Psychopathology University of Groningen Groningen the Netherlands; ^2^ Department of Clinical Psychological Science Maastricht University Maastricht the Netherlands; ^3^ Department of Eating Disorders, Accare Child and Adolescent Psychiatry University of Groningen Groningen the Netherlands

**Keywords:** adolescents, body functionality, body image, eating disorders, intervention

## Abstract

**Objective:**

Negative body image is thought to play an important role in the onset and maintenance of anorexia nervosa and bulimia nervosa. The intervention Expand Your Horizon (EYH), which is focused on increasing functionality appreciation, is being investigated as a potential approach for improving body image. This study investigated the themes that were identified when adolescent girls with an eating disorder are asked to describe their body functionality within the context of EYH.

**Method:**

Fifty‐eight girls with an eating disorder received the EYH intervention and wrote about everything their body can do and why these functions are meaningful to them. The qualitative data were analyzed via Thematic Analysis.

**Results:**

We identified five themes from the data: body functions as a means to experience and regulate emotions; body functions as a vehicle to form social connections; body functions as a means to experience independence and identity; how body functions are intertwined with the eating disorder; and the aesthetic body.

**Discussion:**

This study highlights the importance of body functionality in the experiences and perceptions of adolescent girls with eating disorders. The findings support the idea that adolescents with eating disorders are able to appreciate their bodies for what they can do rather than focusing solely on their appearance.

**Summary**

- In this study we investigated the themes that were identified when young women with an eating disorder were asked to focus on their body functionality.
- We found that adolescents with eating disorders are able to appreciate their bodies for what they can do rather than focusing solely on appearance, which may be associated with reduced self‐objectification and a more positive body image.

## 1 Introduction

Eating disorders are life‐threatening illnesses with severe medical, psychological, and social consequences (Keski‐Rahkonen and Mustelin 2016). Negative or disturbed body image is one of the core characteristics of anorexia nervosa and bulimia nervosa (American Psychiatric Association 2013) and is thought to play an important role in the onset and maintenance of these eating disorders (American Psychiatric Association 2013; Fairburn et al. 2003). Current treatments for eating disorders try to improve individuals' body image via self‐monitoring, cognitive restructuring and exposure exercises (Jarry and Cash 2011; McLean and Paxton 2019). Unfortunately, eating disorder treatments appear limited in their effectiveness and even after successful treatment, relapse rates are high (Berends et al. 2018; Murray et al. 2019; Olmsted et al. 2015; Ricca et al. 2010; Sala et al. 2023; van den Berg et al. 2019). Focusing on improving body image seems key to further improving the treatment effectiveness for eating disorders in the long term (Cornelissen and Tovée 2021; Dingemans et al. 2016), considering that negative body image after treatment was found to be related to a higher risk of relapse (Danielsen and Rø 2012; Keel et al. 2005). One approach to improve body image may be increasing the appreciation of the functionality of one's body, which is considered a core component of positive body image (Swami et al. 2020; Tylka and Wood‐Barcalow 2015).

*Body functionality* concerns everything that the body can do, including physical activities (e.g., exercising), creative activities (e.g., painting), communication with others (e.g., via body language), self‐care (e.g., showering), internal processes (e.g., digestion), and bodily senses and sensations (e.g., smell) (Alleva et al. 2015). *Functionality appreciation* is defined as “appreciating, respecting, and honouring the body for what it is capable of doing, extending beyond mere awareness of body functionality” (Alleva et al. 2017, 21). In qualitative research, participants with a positive body image expressed an appreciation of their body functionality (Frisén and Holmqvist 2010; Wood‐Barcalow et al. 2010). In addition, women who had experienced disordered eating in the past, reported that learning to appreciate their body functionality was a key factor in the transition towards a more positive body image (Alleva, Tylka, et al. 2023). Furthermore, in a systematic review of quantitative research, functionality appreciation was positively associated with other facets of a positive body image and fewer eating disorder symptoms (Linardon et al. 2023).

Why functionality appreciation could be an important key to body image improvement might be explained by objectification theory (Fredrickson and Roberts 1997). In Western society and even beyond Western cultures, girls and women are often valued and evaluated predominantly based on their looks (Gattino et al. 2023). Self‐objectification occurs when someone values their own body—and the self as a whole—based on their physical appearance (Heflick et al. 2011). Self‐objectification can lead to body surveillance and body shame, which in turn may contribute to disordered eating and negative body image (Calogero 2012; Fredrickson and Roberts 1997; Jongenelis and Pettigrew 2020). Improving the appreciation of body functionality could broaden the perception towards valuing the functions of the body, which might lower self‐objectification. Another theoretical framework that might be relevant is the transdiagnostic model for eating disorders (Fairburn et al. 2003). According to this model, the core factor involved in the onset and maintenance of eating disorders is an overvaluation of eating, shape, and weight, meaning that someone judges their self‐worth largely in terms of eating, shape or weight, and the ability to control them. Again, appreciation of one's body functionality might lead to improvements in body image, because individuals learn to broaden their perspective on the self by appreciating their body for what it can do. Alleva et al. (2015) developed the *Expand Your Horizon* (EYH) programme to increase functionality appreciation. In EYH, participants practice focusing on and appreciating the functionality of their body using three online structured writing exercises of 15 min each. Women with a negative body image who participated in the programme experienced lower levels of self‐objectification and higher levels of appearance and functionality satisfaction than participants in the active control condition (Alleva et al. 2015). Other studies also reported positive effects of EYH, for example on weight bias internalization and self‐compassion in women with weight bias internalization (Davies et al. 2022) and among mothers of young children (Granfield et al. 2023). A systematic review identified EYH as the intervention with the best evidence of effectiveness at promoting positive body image in adults (Guest et al. 2019). Besides the quantitative analyses of the effectiveness of EYH, some studies have also reported qualitative analyses of the content of participants' responses to the writing exercises. These studies identified several important themes, such as the enjoyment of body functions, a positive body‐self connection, the body in relation to important others, the resilient body and the aesthetic body. These qualitative findings inform the quantitative data showing that writing appreciatively about one's body functionality (within the context of EYH) can contribute to a more positive body image (Alleva, Atkinson, et al. 2023a; Alleva et al. 2019). EYH already showed promising findings across several populations (Guest et al. 2019). However, to the best of our knowledge, EYH has not yet been investigated in clinical groups of individuals with an eating disorder nor in young individuals (Guest et al. 2019), despite the fact that adolescence is a critical period for the development of body image problems (Möllmann et al. 2023) and that eating disorders are highly prevalent in this age group (Solmi et al. 2022). Given the persistence of negative body image and the need for more effective eating disorder treatments, it is important to investigate EYH in young individuals with an eating disorder. In another paper reporting the quantitative results of the current study, we found that writing about body functionality led to an increase in positive body image, but not to a decrease in negative body image (Glashouwer et al. 2025). In this qualitative study, we aimed to identify the overarching themes that capture how female adolescents describe and reflect on their bodies in terms of what they can do rather than how they look, based on their responses across the EYH writing exercises.

## 2 Method

### 2.1 Participants

In total, 58 female participants (*M*_age_ = 16.76, SD = 1.81) were recruited via the Department of Eating Disorders of Accare (a facility for child and adolescent psychiatry in the Netherlands). The inclusion criteria were: (a) identifying as female, (b) aged 14–23 years old (we included a wide age range for feasibility reasons, aiming to include all eligible participants from our healthcare institute), (c) currently receiving treatment (outpatient therapy, e.g., cognitive behavioral therapy), (d) being fluent in Dutch, and (e) being formally diagnosed with an eating disorder (i.e., anorexia nervosa, bulimia nervosa or an eating disorder not otherwise specified) by a therapist based on the DSM‐5 criteria and the use of the child version of the Eating Disorder Examination (Bryant‐Waugh et al. 1996). Most participants followed higher secondary education (*n* = 32), followed by middle secondary education (*n* = 12), pre‐vocational secondary education (*n* = 6), higher vocational education (*n* = 3) and university (*n* = 2). Three participants did not report their education level. Participants were undergoing treatment for AN of the restrictive type (*n* = 18), AN of the purging type (*n* = 3), atypical AN (*n* = 4), BN (*n* = 6), or another specified eating disorder with features of AN or BN (*n* = 24); the specific classification of three participants was unknown because some patient records lacked accurate data for formal verification of their diagnoses by therapists. Given that Body Mass Index (BMI; weight/height^2^) in children changes with age, we calculated Adjusted BMI scores based on participants' self‐reported age and weight ([actual BMI/The 50th percentile of BMI for age and gender] × 100) (Le Grange et al. 2012). We obtained the 50th percentile of BMI for age and gender from the Netherlands Organization for Applied Scientific Research (TNO 2022). The average adjusted BMI was calculated for 57 of the 58 adolescent girls that participated in the study; one participant did not report her weight. The average adjusted BMI was 95.36% (SD = 13.81, range = 69.19–132.84).

### 2.2 Intervention

In this study we used a one‐session version of EYH as applied in previous studies (e.g., Alleva et al. 2024, 2016). In brief, participants were instructed to write about everything their body can do and why these functions are meaningful to them. If they needed inspiration, they could look at a list with examples of body functions, categorized by domain. The writing exercise consisted of three subparts, each focused on two domains of body functions (see Supporting Information material for the instructions): (1) bodily senses and movement, (2) internal processes and creativity, and (3) self‐care/daily routines and communication. The writing prompts were based on those used in the one‐session EYH body functionality intervention in previous studies (Alleva et al. 2024, 2016). The writing exercise (including reading the instructions) lasted approximately 20 min and was administered on a computer using the program Qualtrics (Qualtrics 2018).

### 2.3 Procedure

The present study is part of a randomized controlled trial to evaluate the short‐term effectiveness of EYH (one‐session version) on body image in adolescent girls with an eating disorder. Quantitative results of the study were reported in a previously published paper (Glashouwer et al. 2025). The study was approved by the Medical Ethical Committee of the University Medical Center Groningen (NL64270.042.18) and was preregistered in the Dutch Trial Register (NTR7704). Individuals who received treatment in the Department of Eating Disorders of Accare and who met the inclusion criteria were informed about the study by their practitioner. All participants gave informed consent before participating in the study. Parents/guardians provided informed consent for participants under age 16. The study was performed at a location of Accare. Participants received a voucher worth €8 for participating in the study.

Participants either received the functionality writing exercise or a control exercise. The control exercise consisted of three short writing assignments about details that participants observe during a route they often take (Alleva et al. 2016). The study made use of a cross‐over design: after completing the first condition (i.e., the EYH writing exercise or the control exercise), the participants also received the other condition, so eventually all participants completed the EYH writing exercise. The present study only reports on the qualitative data of the EYH writing exercise responses.

### 2.4 Reflexivity Statements

Thematic analysis is grounded within the participants' data (bottom‐up), yet can never happen in a “theoretical vacuum” (Braun and Clarke 2006). The interpretation of the data is potentially influenced by our own backgrounds and experiences namely, the first author (S.W.) is a 29‐year‐old white cisgender woman, born and living in the Netherlands. She is doing a Postdoc in psychology and studies the role of disgust in individuals with anorexia nervosa. The second author (J.M.A.) is a 38‐year‐old cisgender woman with a mixed cultural background, born in Canada and currently living in the Netherlands. She is an assistant professor in body image, with a focus on positive body image and body functionality. The third author (K.A.G.) is a 42‐year‐old white cisgender woman, born and living in the Netherlands. She works as a professor by special appointment with a focus on eating disorders in youth.

### 2.5 Qualitative Analysis

We used thematic analysis according to the steps of Braun and Clarke (2006). We chose thematic analysis because it enabled us to explore and interpret the meanings, patterns, and depth of adolescents' reflections on body functionality, extending beyond the literal content of their writing (Braun and Clarke 2006). Specifically, we used “coding reliability thematic analysis” given that we involved multiple team members and the calculation of inter‐rater agreement for codes (see Braun and Clarke (2019)). Our analysis focused on patterns of meaning in participants' reflections that emerged beyond the specific examples or instructions provided in the writing prompts (cf. Braun and Clarke 2006). First, S.W. read the responses to the writing exercises multiple times to familiarize herself with the data. While reading, she made notes about potential patterns in the data and interesting features of the data, generating initial codes. K.A.G. and J.M.A. also independently read the writing exercise responses from five randomly selected participants and made notes while reading. Second, the three researchers discussed the data, their notes and the initial codes to generate an initial set of themes. Third, S.W. reread the data to check whether the initial themes were complete and reflected the data. Fourth, K.A.G. and J.M.A. read the writing exercise responses of three additional randomly selected participants and discussed their reflections with S.W. to create a final set of themes. Subsequently, S.W. coded the entire dataset based on these themes. K.A.G. also coded a random subset of one‐third of the data to calculate interrater reliability. The initial interrater agreement was *ϰ* = 0.62 (range: 0.45–0.79), reflecting a weak to moderate level of agreement. After discrepancies were discussed in the team, the average kappa increased to *ϰ* = 0.96 (range: 0.77–1.00), reflecting strong final interrater reliability (McHugh 2012).

## 3 Results

Below we describe the five themes and sub‐themes from participants' responses to the EYH writing exercise (see Table 1 for an overview of the themes and additional example quotes). The overarching themes were identified from across the three subparts of the writing exercise: (1) bodily senses and movement (average 123 words, min 37–max 257, in the written entries), (2) internal processes and creativity (average 127 words, min 29–max 276, in the written entries), and (3) self‐care/daily routines and communication (average 131 words, min 49–max 256, in the written entries).

**TABLE 1:** An overview of the themes, their descriptions and example quotes.

Theme	Description	Example quote
1. Body functions as a means to experience and regulate emotions	Participants described that due to their body functions they experience *positive emotions* (sub‐theme 1a) (e.g., as a result of spending time with a friend) and pleasure (e.g., by enjoying nature and the smell of food). Participants described their body functions in relation to their *negative emotions* (sub‐theme 1b) as a double‐edged sword: On the one hand, participants expressed difficulties with experiencing and expressing negative emotions. On the other hand, they also reported positive sides of expressing negative emotions (e.g., it provides relief). Participants also expressed the importance of *body functions in helping to regulate emotions* (sub‐theme 1c) (e.g., via exercise, communication with others and creative activities).	“I can talk about my feelings with others, so I don't always have to keep everything to myself. This makes me calmer. When I am sad or high in emotion, tears come and I cry, so it is clear to other people which emotion I am experiencing. I don't have to tell them anything and they know I'm not feeling well.” (participant 5) “I cry quite easily, but I don't like to show that to others because I don't want people to think I'm weak.” (participant 20) “Creative activities give me the opportunity to process my emotions or to completely clear my head.” (participant 7)
2. Body functions as a vehicle to form, experience, and elicit meaningful social connection	Participants described the importance of various body functions as a means to form and experience meaningful *social connections* (sub‐theme 2a) (e.g., sharing a meal with a parent, falling in love with a new partner) as well as to elicit meaningful social connection and *support towards and from* others (sub‐theme 2b and 2c) (e.g., crying can elicit empathy and a hug, expressing anger can elicit problem‐solving from a friend).	“I like chatting and drinking tea with friends or my mother. We laugh a lot and that makes us very happy. If we don't feel so good, we can turn to each other and that's nice. I also have the right people around me who I can rely on and who will put an arm around me when things go wrong.” (participant 9) “My body makes it possible to hug friends and family. I can talk to my parents when I'm going through a difficult time.” (participant 33) “I can talk, smile, listen to others and try to be there for them when they are having problems. Without that, I would never be able to have a good relationship with my friends.” (participant 11)
3. Body functions as a means to foster, experience, and express independence and identity	Participants described the importance of their body functions as a means to foster and experience *independence* (sub‐theme 3a) (e.g., autonomy, being able to do what you want, reaching goals in life), and embodying and creating the own *identity* (sub‐theme 3b) (i.e., becoming a mother).	“I think it is important that I can see, taste, hear, smell and feel, because then you can experience (new) things. You can discover and view. You can do anything. Without these things you wouldn't be able to live normally and that is ultimately what everyone wants.” (participant 12) “My body can have a baby so I can have a family that I love.” (participant 39)
4. Body functions are intertwined with the eating disorder	Participants described the relationship between their eating disorder a body functionality in three ways: (1) *current barriers and costs due to the ED* (sub‐theme 4a) (e.g., less energy, no longer doing a favorite hobby, experiencing difficulties in connecting with others); (2) *regaining of previous function after partial recovery of the ED* (sub‐theme 4b) (e.g., being able to bike ride again); and (3) *the way in which body functions help in the recovery of ED* (sub‐theme 4c) (e.g., getting sufficient rest and sleep helps to “fight” the eating disorder).	“It is also very important for me to be able to have children later. I know that because I have not had my period for a long time, there is a chance that I will never be able to have children. That hurts me very much.” (participant 12) “I sleep better without thinking about how much you ate or what you are going to eat tomorrow.” (participant 14) “I can also do strength training, which reminds me again and again what my body can do and that I can also become stronger instead of just focusing on my appearance. This also helps me in recovery, so exercise can be therapeutic.” (participant 45)
5. The aesthetic body: Appreciation and concerns	Participants described their appearance in *positive terms* (sub‐theme 5a) (e.g., what they felt satisfied with, what they appreciated) and/or in *negative terms* (sub‐theme 5b) (e.g., disgust for the appearance of their body).	“I really enjoy exercise and it gives me peace and it helps you build muscles and improve your figure.” (participant 16) “I can see and I really like that, but it is sometimes very difficult because I also see myself all the time.” (participant 20)

### 3.1 Body Functions as a Means to Experience and Regulate Emotions

Almost all (54/58) participants mentioned body functions *as a means to experience and regulate emotions*. Their reflections revealed that body functions are closely tied to both positive and negative emotional experiences, as well as to strategies for emotional expression and regulation. Most participants (50/58) reported that because of their body functions they are able to experience *positive emotions*, *pleasure, and enjoyment* (sub‐theme 1a) and positive sensations like joy and pleasure were often linked to physical experiences (e.g., enjoying nature or going for a walk). One participant emphasized that through her body functions she can also make other people experience positive emotions: “When I perform [at musical concerts], I receive the feedback that I make people happy and that they enjoy it, and that makes me very happy! I'm glad my body can do all these things” (participant 9). Remarkably, experiencing positive emotions was a central part in participants' responses. They often linked positive emotions to other themes, such as social connection, and wrote about positive emotions throughout the writing exercise, extending beyond assignment‐specific subcomponents. *Negative emotions* (sub‐theme 1b, 19/58) were on the one hand described as unpleasant, but on the other hand participants also described they felt relieved after expressing these emotions (e.g., “I can cry. There are times when I can't cry, but when I can, it always brings relief.”; participant 11). Over half of the participants (35/58) mentioned physical activity or creative activities as a way to express and *regulate their emotions* (sub‐theme 1c) (e.g., “I can dance with my body and in this way, I can release myself from different emotions”; participant 18).

### 3.2 Body Functions as a Vehicle to Form, Experience, and Elicit Meaningful Social Connection

Most of the participants (45/58) described their body functions *as a vehicle to form meaningful social connections* (sub‐theme 2a), emphasizing the role of bodily functions and actions in forming relationships. Bodily functions and behaviors facilitated bonding, while emotional expressions like crying often led to supportive responses from others, reinforcing social connection. One participant wrote, “all these functions are important to me because I link them with the word love, and the feeling of loving and being loved” (participant 2). In their responses, participants made a distinction between *providing support towards others* (sub‐theme 2b, 16/58) by means of their body functions (e.g., “my body allows me to hug people: this is important to me because in this way I can show people that I care about them”; participant 10) and *receiving support from others* (sub‐theme 2c, 16/58) (e.g., “sometimes you just need that touch. Just that hug so you know they are there. That is very important so that I don't feel alone”; participant 54). Note that while participants were prompted to describe body functions in relation to communication with others, this theme transcended across the subcomponents of the writing exercise and was identified consistently across all three writing prompts.

### 3.3 Body Functions as a Means to Foster, Experience, and Express Independence and Identity

The majority of the participants (38/58) described their body functions *as a means to experience and express independence and identity*, which they also connected to social connection and the experience of positive emotions throughout the assignment's subcomponents. This theme reflects how physical capabilities, including sensory experiences, allow individuals to participate in meaningful activities, to make their own choices, and to connect with aspects of their personal identity. More than half of the participants (31/58) positively valued the importance of their body functions for experiencing a sense of freedom and autonomy to perform their daily activities (e.g., “through physical activity and exercise I can cycle to school every day, walk my dog, do fun things with friends and my boyfriend. I also get pleasure from this”; participant 24). Some of them emphasized the role of their senses in being *independent* (sub‐theme 3a), which they linked to experiencing pleasure (e.g., “I can hear, so I can listen to my favourite music and musicals. I can see, so I can watch nice series or nice movies”; participant 15). Several participants (13/58) highlighted the role of their body functions in realizing their *identity of becoming a mother* (sub‐theme 3b) (e.g., “My body can carry and grow a baby, so I can one day make my desire to have children come true”; participant 5).

### 3.4 Body Functions Are Intertwined With the Eating Disorder

About a third of the participants (21/58) described their body functions *in relation to their eating disorder*. The eating disorder was described as limiting physical and social activities, while recovery brought back lost abilities. Moreover, supporting physical health was perceived as a key part of recovery. Twelve participants mentioned a *loss of functions* (sub‐theme 4a) or an inability to perform certain activities due to their eating disorder (e.g., “I always played volleyball a lot, but I stopped because of my anorexia”; participant 9). One participant expressed that because of the eating disorder she lost her pleasure in activities that she previously enjoyed: “Before I had an eating disorder, I really enjoyed baking cakes, but now that I haven't had a piece of cake in years, it's not much fun because I'm far too afraid that I might want something and regret it later” (participant 20). Other participants (12/58) valued *regaining certain functions* (sub‐theme 4b) after previous loss due to their eating disorder, such as one participant who described: “If you have enough energy, your mood also improves and you feel better about yourself” (participant 43). Lastly, a few participants (6/58) described how their *body functions helped them with recovering* (sub‐theme 4c) from the eating disorder (e.g., “By sleeping well you are more rested and you can fight the eating disorder better”; participant 31).

### 3.5 The Aesthetic Body: Appreciation and Concerns

Several participants (10/58) described their appearance in response to the functionality writing exercise, despite it not being mentioned in the instructions. Participants described mixed feelings about their appearance, reflecting both positive and negative appreciation of their appearance. Seven of them wrote about growing their nails, hair, looking good and gaining muscular strength in a *positive way* (sub‐theme 5a) (e.g., “I also think that keeping myself clean and dressed up is important to think better about myself and it also ensures that I care less about what others think”; participant 58). Only three participants wrote more *negatively* (sub‐theme 5b) about their appearance and expressed concerns about the way they look (e.g., “I think my body is ugly and I'm ashamed of it”; participant 11).

## 4 Discussion

This study examined the themes that were identified when adolescent girls with an eating disorder write about body functionality within the context of EYH. We identified three themes that were described by the majority of participants: body functions as a means to experience and regulate emotions; body functions as a vehicle to form social connections; and body functions as a means to experience independence and identity. Two themes were identified among a minority of participants: how body functions are intertwined with the eating disorder, and the aesthetic body.

In the first theme almost all of the participants emphasized that their body functions are important for experiencing positive emotions. Participants also linked experiencing positive emotions to the other themes of being independent (theme 3) and to social connections, by making other people experience positive emotions (theme 2). Some participants indicated that they find dealing with negative emotions challenging, which was also one of the themes emphasized by women who completed EYH after having undergone bariatric surgery (Alleva et al. 2023a). These findings are in line with prior studies that reported emotion regulation difficulties in patients with eating disorders (Haynos and Fruzzetti 2011; Oldershaw et al. 2015) and with the transdiagnostic model for eating disorders in which mood intolerance is one of the core components involved in the maintenance of eating disorders (Fairburn et al. 2003).

In the second theme, participants positively emphasized the importance of their body functions for forming meaningful relationships and for support. Previous qualitative studies identified having a supportive social environment with friends and family members who accept you for who you are as a crucial element for developing a positive body image (Alleva, Tylka, et al. 2023; Gattario and Frisén 2019). Having a supportive social environment might be especially important for adolescents with an eating disorder, since support from family and peers is associated with fewer eating disorder symptoms (Kirsch et al. 2016; Stern et al. 2023).

The third theme reflects the enjoyment of body functions by promoting autonomy and being able to express one's identity (e.g., the wish to become a mother later in life). This theme was not found in the previous qualitative studies in which participants received the EYH programme (Alleva, Atkinson, et al. 2023a; Alleva et al. 2019). One likely explanation for this difference in results between our sample of female adolescents with an eating disorder and the adult samples including both men and women in the studies by Alleva et al. (2019, 2023a), can be the differences in age and gender of the participants. Both age and gender are associated with self‐reported levels of body satisfaction (Hockey et al. 2021). Furthermore, given the broad age range of participants, it is important to consider that developmental differences within adolescence (e.g., social or emotional maturity) may have influenced the responses of the participants. Adolescence and young adulthood are a time in which individuals aim for independence and develop their identity and ideas about the future self (Spear and Kulbok 2004). A focus on the appreciation of bodily functions and linking this to independence might support the formation of an identity that extends beyond the eating disorder. Therefore, it could be valuable to explicitly add the role of bodily functions in supporting independence and identity to the instructions of EYH.

The fourth theme reflects both health concerns as well as gratitude towards bodily functions. On the one hand, participants wrote about function loss due to their eating disorder; on the other hand, they described how their body functions helped them with recovering from their eating disorder. The latter is in line with the gratitude model of body appreciation (Homan and Tylka 2018): writing about gratitude for your body could lead to improvements in body image by shifting the focus away from appearance as a source of self‐worth (Dunaev et al. 2018). In future work, we suggest investigating whether adding explicit writing instructions to the EYH protocol on how body functions can help in recovering from an eating disorder might strengthen its effects.

The fifth theme, the aesthetic body, refers to writing about body functionality in relation to one's appearance. Notably, this theme was less salient than in a prior study among participants who underwent bariatric surgery (Alleva, Atkinson, et al. 2023a). In addition, it is reassuring to find that only three participants wrote about their appearance in negative terms. This suggests that EYH may help participants focus on the appreciation of body functionality. Taking into consideration that three participants did write about their appearance in a negative way, future functionality interventions could include clearer instructions to focus on body functions only, to help participants distinguish functionality from appearance.

Taken together, the qualitative results inform the quantitative results of this study, which found that the functionality writing exercise improved participants' scores on positive body image in comparison with the control condition (Glashouwer et al. 2025). The qualitative results indicate that the writing exercise might be able to broaden the perspective of adolescents with an eating disorder away from purely evaluating themselves based on their appearance and their ability to control their shape and weight (Fairburn et al. 2003). Both qualitative and quantitative (Glashouwer et al. 2025) results reflect that EYH may be a promising approach to explore in the context of body image work with young women with an eating disorder.

A limitation of this study is that we cannot distinguish between different eating disorders classifications. Some themes might be more salient for individuals with a particular eating disorder than others; for example, the loss of bodily functions (e.g., menstrual cycle) might be particularly relevant to low body weight in adolescents with anorexia nervosa. Future research could analyze the results of the writing exercises in relation to the DSM‐5 classification to investigate whether certain themes are more salient for specific eating disorder classifications. Furthermore, although the themes were identified across subcomponents of the writing exercise, the results may have been shaped by the specific prompts provided, which could have influenced the content of participants' responses. Yet, we deemed it necessary (cf. Alleva et al. 2015) to provide examples across domains, to ensure that participants had a clear understanding of body functionality, in a holistic sense, prior to writing. A last potential limitation is the use of an open‐response survey, which may have limited the depth of participants' reflections compared with interactive methods like in‐depth, one‐on‐one interviews. Future research could explore how treatment context may shape participants' engagement with the writing tasks, as therapeutic background might influence interpretation and response. Moreover, future research could explore the use of artificial intelligence tools to complement thematic analysis, while carefully considering data confidentiality.

## 5 Conclusions

In this study, we investigated the themes that were generated when female adolescents with an eating disorder were asked to describe their body functionality within the context of EYH. The identified themes demonstrate that adolescents with eating disorders are able to appreciate their bodies for what they can do rather than focusing solely on appearance. For future studies, it could be valuable to specifically instruct adolescents with an eating disorder to focus on how bodily functions are important for the formation of identity and how they can help in eating disorder recovery. More specifically, future research could examine how the reflection of certain themes in participants' answers, as well as the length or depth of their responses, relates to treatment outcomes. Such analyses could help identify which aspects of the intervention are most critical for promoting positive change. In conclusion, this study highlights the potential benefits of focusing on body functionality in the treatment of eating disorders, specifically through the EYH writing exercise.

## Author Contributions

**Stella Weiland:** data curation, formal analysis, writing – original draft; **Jessica M. Alleva:** conceptualization, methodology, formal analysis, writing – review and editing. **Klaske A. Glashouwer:** conceptualization, methodology, investigation, formal analysis, supervision, writing – review and editing.

## Ethics Statement

The study was approved by the Medical Ethical Committee of the University Medical Center Groningen (NL64270.042.18) and was preregistered in the Dutch Trial Register (NTR7704). All participants gave informed consent before participating in the study. In case participants were below the age of 16, their parents/guardians provided informed consent.

## Conflicts of Interest

The authors declare no conflicts of interest.

## Supporting information


**Data S1:** eat70006‐sup‐0001‐supinfo.docx.

## Data Availability

The data that support the findings of this study are available from the corresponding author upon reasonable request.
