# Supplementary material for: Expand Your Horizon: A Qualitative Analysis of How Adolescent Girls With an Eating Disorder Describe Their Body Functionality
Source: Int J Eat Disord. 2025 Nov 18;59(3):510–8. doi: 10.1111/eat.70006 (PMC12979954; doi:10.1111/eat.70006)
Supplement: Supplementary file 1 — Data S1: eat70006‐sup‐0001‐supinfo.docx. [file EAT-59-510-s001.docx]

**Instructions Writing Exercise Experimental Condition**

When we think about our body, we usually think about what our **body looks like** (e.g., your weight or shape). In a moment you will learn to see your body in a different way. Instead of focusing on the appearance of your body, you will learn to focus **on everything your body can do**. We call this **body functionality.**

Below you will find a list of bodily functions that other girls have written about. Read them carefully. You probably already thought of some features; you may not have considered other features yet: **Body functions in the field of senses and sensations** Sight Taste Hearing Smell of Touch Experiencing pleasure Feeling emotions **Body functions in the field of physical activity and movement** Running Jumping Walking Stretching Flexibility Physical coordination Agility Balance Strength Endurance Energy level Reflexes Sports (e.g. football, swimming, Zumba) Yoga Climbing Cycling **Body functions in the field of health** Healing (e.g. recovering from a cold, of a wound) Digesting food Absorbing vitamins Being able to have a baby Growing (e.g. hair, nails, new skin cells) Regulation (e.g. temperature, hunger, thirst) General recovery (e.g. while sleeping) Removing waste from the body (e.g. through the liver) Breathing **Body functions in the area of ​​creativity** Dancing Painting Drawing Building Modeling Sculpting Writing Singing Playing an instrument Reading Photography Gardening **Body functions in the area of daily routines** Sleeping/napping Eating Drinking Cooking Body care (e.g. showering, taking a bath) **Body functions in the areas of relationships with others and communicating** Talking Body language Facial expressions (e.g. smiling) Hugging Kissing Crying Shaking hands Making eye contact A shoulder to cry on Giving or receiving a massage Writing a letter Hugging

That was quite a list! Now take a moment to think about the importance of these bodily functions for your life. What do these features mean to you? For example, what would your life be like if you couldn't cycle or walk to school or work? Or how boring would life be if you couldn't watch your favorite series or listen to music? Another example: what if you couldn't communicate with your body (e.g., giving hugs, or chatting with others)? Or what if you couldn't use your body for creativity (e.g., painting, dancing)? Body functions can be simple (e.g., running) or complex (e.g., recovering from the flu), but they are all important for living a normal and worthwhile life.

**Writing assignment #1**Before, I asked you to think about the different functions of your body. In this writing assignment, I would like to ask you to describe in more detail what your body can do.

Take your time; think as freely as possible and consider the different things your body can do. In this first writing assignment, you will focus only on bodily functions related to (1) your **body's senses and sensations** (e.g., vision, experiencing pleasure) and (2) **physical activity and movement** (e.g., going for a walk, dancing). If you need inspiration during the assignments, you can always look at the list of bodily functions (see example list above).

When writing about the functions of your body, it is important that you also think about what these functions mean to you. Ask yourself "Why are these features important to me?" Don't forget that every simple or more complex bodily function plays an important role in our lives, even if we hardly think about it.

This assignment takes a few minutes, you will be notified when the time is up. Try to fill the entire time with writing. Finally: Your writing assignment will be treated confidentially and anonymously. Don't worry about spelling, sentence structure, or grammar. Different brains can think different things. Your writing assignment is therefore unique in any case. So there are no 'right' or 'wrong' descriptions.

What can you do with your body in terms of 1) **senses and sensations** and 2) **physical activity and movement**? What does this mean to you?

**Writing Assignment #2**In your second writing assignment, you will focus on your bodily functions related to (1) **health** (e.g., healing, digesting food) and (2) **creative activities** (e.g., drawing, writing). As in your first writing assignment, it is important that you think about what these different functions mean to you. Ask yourself, "Why are these features important to me?"

What can you do with your body in terms of 1) **health** and 2) **creative activities**? What does this mean to you?

**Writing Assignment #3**
In your third writing assignment, you will focus on the bodily functions related to (1) **daily routines** (e.g., showering and sleeping) and (2) **relationships with others and communication** (e.g., hugging, kissing). As you write, remember to think about what these features mean to you, and to ask yourself "Why are these features important to me?"

What can you do with your body in terms of 1) **daily routines** and 2) **relationships with others and communication**? What does this mean to you?
